# Risk Factors of Progression in Low-tumor Burden Follicular Lymphoma Initially Managed by Watch and Wait in the Era of PET and Rituximab

**DOI:** 10.1097/HS9.0000000000000861

**Published:** 2023-04-26

**Authors:** Cyrielle Rodier, Lukshe Kanagaratnam, David Morland, Adélie Herbin, Amandine Durand, Adrien Chauchet, Sylvain Choquet, Philippe Colin, René Olivier Casasnovas, Eric Deconinck, François Godard, Alain Delmer, Cédric Rossi, Eric Durot

**Affiliations:** 1 Department of Hematology, University Hospital of Reims, Hôpital Robert Debré, Reims, France; 2 UFR Médecine, Reims, France; 3 Department of Research and Innovation, University Hospital of Reims, Hôpital Robert Debré, Reims, France; 4 Médecine Nucléaire, Institut Godinot, Laboratoire de Biophysique, UFR de Médecine, Université de Reims Champagne-Ardenne, and CReSTIC (Centre de Recherche en Sciences et Technologies de l’Information et de la Communication), EA 3804, Université de Reims Champagne-Ardenne, Reims, France; 5 Department of Hematology, University Hospital F. Mitterrand and Inserm UMR 1231, Dijon, France; 6 Department of Hematology, University Hospital of Besançon, France; 7 Department of Hematology, APHP, Groupe Hospitalier Pitié-Salpêtrière, Paris, France; 8 Department of Oncology, Clinique Courlancy, Reims, France; 9 Médecine Nucléaire, Centre Georges-François Leclerc, Dijon, France

## Abstract

Patients (pts) with asymptomatic low-burden follicular lymphoma (FL) are usually observed at diagnosis. Time to lymphoma treatment (TLT) initiation can however be very heterogeneous and risk factors of progression are poorly studied. Our study evaluated 201 pts with grade 1–3a low-tumor burden FL diagnosed in four French centers between 2010 and 2020 and managed by a watch and wait strategy in real-life settings. After a median follow-up of 4.8 years, the median TLT was 4.2 years (95% confidence interval, 3.1-5.5). On multivariate analysis, elevated lactate dehydrogenase (hazard ratio [HR] = 2.2; *P* = 0.02), more than 4 nodal areas involved (HR = 1.7; *P* = 0.02) and more than 1 extranodal involvement (HR = 2.7; *P* = 0.01) were identified as independent predictors of TLT. The median TLT was 5.8 years for pts with no risk factor, 2.4 years for 1 risk factor, and 1.3 years for >1 risk factors (*P* < 0.01). In a subanalysis of 75 pts staged with positron emission tomography-computed tomography (PET-CT), total metabolic tumor volume (TMTV) ≥14 cm^3^ and standardized Dmax (reflecting tumor dissemination) >0.32 m^−1^ were also associated with shorter TLT (HR = 3.4; *P* = 0.004 and HR = 2.4; *P* = 0.007, respectively). In multivariate models combining PET-CT parameters and clinical variables, TMTV remained independent predictor of shorter TLT. These simple parameters could help to identify FL patients initially observed at higher risk of early progression. The role of PET-CT (extranodal sites and PET metrics) in low-burden FL appears promising and warrants further assessment in large cohorts.

## Graphical abstract

**Figure:**
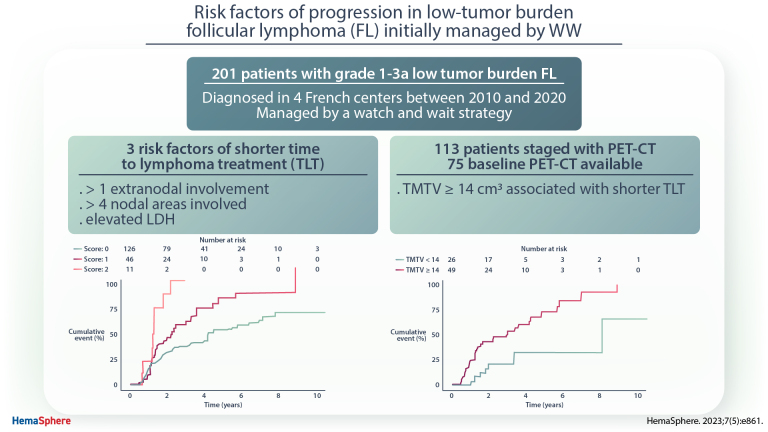

## INTRODUCTION

Follicular lymphoma (FL), the most frequent indolent non-Hodgkin lymphoma (NHL), is a heterogeneous disease, usually incurable.^1,2^ The course of the disease is characterized by repeated relapses and multiple lines of therapies, with increasingly short free intervals, and sometimes histological transformation (HT) into aggressive NHL.^3^ Criteria established by Groupe d’Étude des Lymphomes Folliculaires (GELF), British National Lymphoma Investigation, or Gruppo Italiano Trapianto Midollo Osseo are used to distinguish low and high-tumor burden FL. Anti-CD20 antibodies, in combination with chemotherapy and in maintenance, have improved outcomes.^4,5^ Patients with high-tumor burden FL are usually treated with immunochemotherapy ± maintenance, while watch and wait (WW) until disease progression is a recognized and recommended strategy for low-tumor burden FL patients.^6^ Retrospective and randomized prospective studies in the pre- and post-rituximab era did not found any difference in overall survival (Os) between immediate treatment and initial observation in asymptomatic, non-Bulky FL.^7–9^ However, patients who undergo initial observation can experience heterogeneous clinical course. Some patients can progress early, within 1–2 years, requiring systemic treatment, whereas a proportion of patients remains treatment-free at 10 years, highlighting the need to identify risk factors of progression to adapt the follow-up accordingly.

Many prognostic factors have been identified in FL and several scores have been developed in patients requiring treatment, such as Follicular Lymphoma International Prognostic Index (FLIPI),^10^ FLIPI2,^11^ and PRIMA-prognostic index (PRIMA-PI).^12^ Among other known prognostic factors, [^18^F]fluoro-2-deoxyglucose/positron emission tomography-computed tomography ([^18^F]FDG/PET-CT) parameters, and particularly total metabolic tumor volume (TMTV) at baseline, have been reported as a strong predictor of progression-free survival (PFS) in high-tumor burden FL patients.^13^ Baseline TMTV combined with FLIPI2 allows identifying patients at high risk of early progression.^14^

Conversely, risk factors of progression in initially observed low-tumor burden FL have been poorly studied. The study by Solal-Céligny et al^15^ showed a shorter time to lymphoma treatment initiation (TLT) in patients with involvement of more than 4 nodal sites but no correlation with FLIPI and FLIPI2 scores. Similar correlation was demonstrated in the Danish study, with in addition elevated lactate dehydrogenase (LDH).^16^ Our study aimed to identify risk factors for early progression of low-tumor burden FL managed frontline with WW strategy.

## PATIENTS AND METHODS

### Study population and data collection

We retrospectively analyzed the databases of 4 French centers (Reims, Dijon, Besançon, and Pitié Salpêtrière, Paris) for patients older than 18 years with newly diagnosed grade 1–3a FL between January 2010 and January 2020 and initially managed by a WW strategy. The diagnosis of FL was made in accordance with the World Health Organization classification of malignant lymphoma and confirmed for the majority of the cases by an expert hematopathologist from the Lymphopath network.^17,18^ Patients had to present with low-tumor burden according to GELF criteria. To remind, GELF criteria are bulky mass (over 7 cm), >2 lymph nodes in 3 distinct areas over 3 cm, symptomatic splenomegaly, organ compression by tumor, pleural or peritoneal effusion, B symptoms, elevated LDH, and/or elevated ß2 microglobulin (ß2m). Nevertheless, patients with isolated elevated LDH or ß2m are usually managed by WW and were included in the study. WW was considered to be the initial strategy if no treatment, except diagnosis biopsy, was given during the first 6 months after diagnosis.

Patients with histological grade 3b FL, transformation at the time of diagnosis, nonhematological malignancy diagnosed within 5 years before FL, or other concomitant hemopathy or mixed histology, were excluded.

Baseline clinical and laboratory data were collected from medical records. Initial staging was performed using contrast-enhanced computed tomography or PET-CT according to each center policy. Disease stage was defined according to the Ann Arbor classification.^19^ The number of nodal regions was counted according to the original FLIPI model.^10^ FLIPI score was calculated for patients with available data.

This retrospective study was conducted in accordance with the Declaration of Helsinki and was authorized by the “Commission Nationale Informatique et Libertés” (authorization number 2206749v0), allowing the computerized management of the medical data. The participants were informed of the research purposes and had a right of opposition.

### PET-CT analysis

Baseline PET-CT available for patients from Reims and Dijon were analyzed on a dedicated console system (AW Server, General Electrics, Milwaukee, USA). TMTV was computed by 2 nuclear medicine physicians blinded to patient outcome. TMTV was measured by summing the metabolic volumes of every individual nodal and extranodal lesion, using the 41% thresholding method, as recommended.^20^ A volume of interest was set around each group of lesions as previously described. Only focal uptake was included in the volume measurement of bone marrow (BM) involvement. Spleen was considered as involved in case of focal uptake or diffuse uptake higher than 150% of the liver uptake. The highest maximum standardized uptake value (SUVmax) of the patient over all lesions was also reported.

The distance Dmax between the centers of the 2 most distant lesions, denoted as *A* and *B*, was calculated based on their 3D coordinates (*x*, *y*, *z*) by using the Euclidian formula: Dmax (cm) = √[(*x*_*B*_ − *x*_*A*_)² + (*y*_*B*_ − *y*_*A*_)² + (*z*_*B*_ − *z*_*A*_)²]. This distance was then normalized with the body surface area, given by the Mosteller formula √(weight × height)/3600 (weight in kg and height in cm), yielding the standardized Dmax (SDmax).

The optimal cut offs of Dmax and SDmax for TLT prediction were determined by receiver operating characteristic (ROC) analysis. For TMTV, the recently defined cutoff of 14 cm^3^ was used.^21^

### Statistical analysis

Quantitative variables were expressed as median and range and qualitative variables as number and percentages. The main endpoint was time to progression to lymphoma that required treatment. The date of first lymphoma treatment was used to avoid bias due to different patterns of observation between centers and physicians. TLT was therefore defined as the time from diagnosis to the date of treatment initiation (immunochemotherapy, immunotherapy, or radiotherapy). Data from patients who died before FL progression, did not progress at last follow-up or were lost to follow-up before progression were censored. Cumulative incidence estimates of treatment initiation were calculated. For survival analysis, the Kaplan-Meier method was used, and differences between the curves were tested by log-rank test. Univariate and multivariate analyses were performed using the Cox proportional hazards model. Continuous variables were dichotomized on the basis of usual thresholds. All risk factors with a *P* value <0.10 by univariate analysis were included in the multivariate Cox regression. A manual backward selection procedure was used to define the final model. The results were presented as hazard ratio (HR) and 95% confidence intervals (CI). A *P* value <0.05 was considered statistically significant. Statistical analyses were performed using SAS 9.4 (SAS Institute, Cary, NC, USA) and R version 4.04 (R Core Team 2021, R Foundation for Statistical Computing, Vienna, Austria).

## RESULTS

We identified 884 patients diagnosed with FL on the pathology lists of 4 French centers. We excluded 683 patients because of treatment initiated within 6 months of diagnosis, active concurrent malignancy or other hemopathy, histological grade 3b FL or missing data.

### Patient characteristics

Therefore, 201 patients with newly diagnosed grade 1–3a FL initially observed were identified. The median age at FL diagnosis was 64 years (range, 30–88) and 114 patients (57%) were men. The majority of patients presented with low Eastern Cooperative Oncology Group (ECOG) performance status (0 or 1 in 99%), no B symptoms (97%), normal LDH (93%), hemoglobin >120 g/L (96%), and low or intermediate FLIPI score (86%). More than half of patients were staged with PET-CT. About one fourth of patients had more than 4 nodal regions involved (n = 46) and the same proportion at least one extranodal involvement. The most common extranodal sites were bone and BM (Suppl. Table S1). The patient characteristics are listed in Table 1.

**Table 1:** Baseline Characteristics of Patients With Low-tumor Burden FL

Characteristic	Total (n = 201)
Age (y)	
Median	64 (range 30–88)
>60	110 (55%)
Male	114 (57%)
ECOG performance status ≥1	34 (18%)
Missing	9
B symptoms	6 (3%)
Missing	11
Hemoglobin <120 g/L	8 (4%)
Missing	8
Platelets <150 × 10^9^/L	16 (8%)
Missing	9
Lymphopenia	45 (24%)
Missing	15
Circulating lymphoma cells	7 (5%)
Missing	46
Elevated LDH	14 (7%)
Missing	12
Elevated ß2m	25 (15%)
Missing	32
Serum albumin <3.5 g/dL	6 (5%)
Missing	76
Histological grade	
1–2	170 (92%)
3a	15 (8%)
Missing	16
PET/CT staged	113 (57%)
Number of nodal groups >4	46 (24%)
Missing	7
Extranodal involvement	46 (23%)
≥2	12 (6%)
Missing	4
Bone marrow involvement	14 (30%)
Missing	158
Ann Arbor stage III/IV	119 (59%)
FLIPI	
Low (0–1)	104 (55%)
Intermediate (2)	58 (31%)
High (3–5)	27 (14%)
Missing	12
Initiated treatment	106 (53%)
Histological transformation	23 (11%)
During WW	21 (10%)

ß2m = ß2 microglobuline; ECOG = Eastern Cooperative Oncology Group; FLIPI = Follicular Lymphoma International Prognostic Index; LDH = lactate dehydrogenase; PET/CT = positron emission tomography/computed tomography; WW = watch and wait.

**Figure 1.**
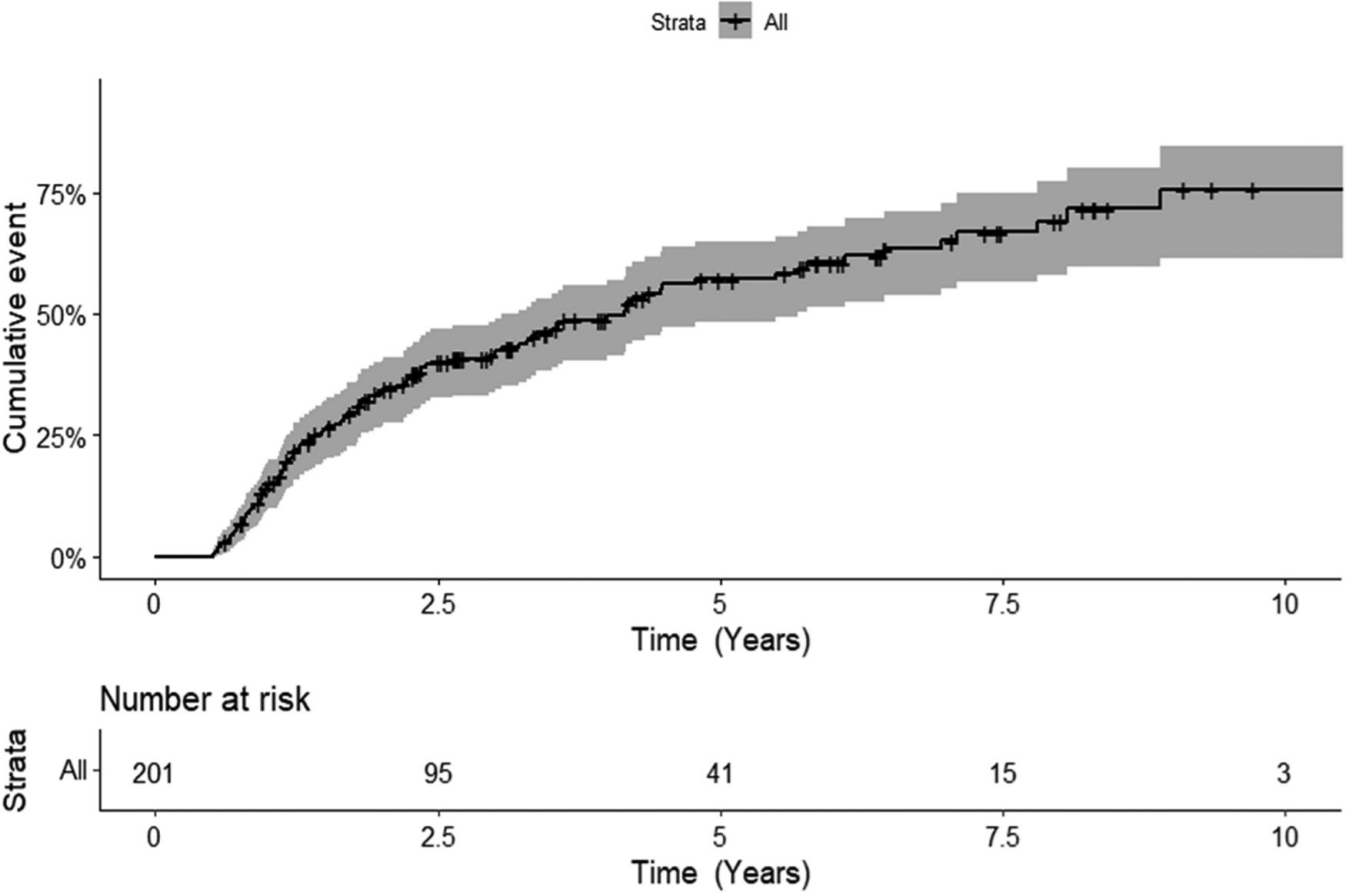
**Cumulative incidence of treatment initiation from the time of FL diagnosis.** FL = follicular lymphoma.

With a 4.8 years median follow-up (95% CI, 3.6-5.9 years), 106 patients (53%) experienced disease progression and were treated, including 21 (10%) with transformation before treatment. The median TLT was 4.2 years (95% CI, 3.1-5.5 years). For the 106 patients who received treatment following disease progression, the median TLT was 1.6 year (95% CI, 1.2-1.9 years). Cumulative incidence of treatment initiation at 1, 2, 3, and 5 years were 15% (95% CI, 10%-19%), 34% (95% CI, 26%-40%), 42% (95% CI, 34%-48%), and 57% (95% CI, 48%-65%), respectively (Figure 1).

First-line therapy consisted of rituximab (n = 77) or obinutuzumab (n = 13) in combination with chemotherapy (CHOP in 90%, bendamustine in 10%) in 85% (n = 90) of cases, radiotherapy alone in 8% (n = 8), rituximab single-agent in 4% (n = 4), and anti-CD20 antibody in combination with lenalidomid in 2% (n = 2). Seventy-three patients (69%) received maintenance therapy by rituximab (n = 61) or obinutuzumab (n = 12).

The 5-year OS was 93% (95% CI, 89%-98%) (Suppl. Figure S1). There were 13 deaths (6%). Four patients died without initiation of treatment (2 of other cancer diagnosed after FL, 2 of unknown causes). Nine patients died after progression: 6 deaths were related to lymphoma (infection in 3 patients, transformed FL in 2, concurrent malignancy and transformed FL in 1), 1 of solid cancer and 2 died of unknown cause.

### Risk factors for progression

In univariate analysis, hemoglobin <120 g/L (*P* = 0.04), elevated ß2m (*P* = 0.05), Ann Arbor stage III/IV (*P* = 0.007), involvement of more than 4 nodal areas (*P* = 0.003), and more than one extranodal site (*P* = 0.005) were significant predictors of TLT (Table 2). Elevated LDH, histological grade 3a and lymphocytes <1 × 10^9^/L had a *P* value <0.10 (*P* = 0.06, 0.07, and 0.08, respectively). Type of extranodal sites (bone, BM, digestive tract, lung/pleura) was not associated with a shorter TLT.

**Table 2:** Impact of Baseline Characteristics on Time to Treatment of Low-tumor Burden FL

Parameter	Univariate Cox Proportional Hazard		Multivariate Cox Regression Analysis	
	HR (95% CI)	*P* Value	HR (95% CI)	*P* Value
Age >60 y	1.0 (0.7-1.5)	0.83		
ECOG performance status >1	0.7 (0.4-1.3)	0.23		
B symptoms	0.3 (0.1-2.0)	0.21		
Lymphopenia	1.5 (0.9-2.4)	0.08		
Circulating lymphoma cells	1.8 (0.7-5.0)	0.25		
Hemoglobin <120 g/L	2.7 (1.1-6.6)	0.04		
Platelets <150 × 10^9^/L	1.2 (0.5-2.6)	0.67		
Elevated LDH	1.9 (1.0-3.7)	0.06	2.2 (1.1-4.3)	0.02
Elevated ß2m	1.7 (1.0-3.1)	0.05		
Albumin <3.5 g/dL	1.0 (0.2-4.2)	0.98		
Histological grade 3a	0.4 (0.2-1.1)	0.07		
Number of nodal groups >4	1.9 (1.2-3.0)	0.003	1.7 (1.1-2.8)	0.02
Extranodal involvement ≥2	2.7 (1.3-5.4)	0.005	2.7 (1.2-5.7)	0.01
Bone marrow involvement	1.2 (0.5-2.7)	0.73		
Ann Arbor stage III/IV	1.7 (1.2-2.6)	0.007		
FLIPI high risk	2.6 (1.5-4.5)	< 0.001		
Year of FL diagnosis	1.0 (1.0-1.1)	0.27		

ß2m = ß2 microglobuline; ECOG = Eastern Cooperative Oncology Group; FL = follicular lymphoma; FLIPI = Follicular Lymphoma International Prognostic Index; HR = hazard ratio; LDH = lactate dehydrogenase.

To avoid any bias in TLT estimates due to possible changes in patient management during the 10-year study period, year of FL diagnosis was tested and was not associated with TLT (*P* = 0.27).

**Figure 2.**
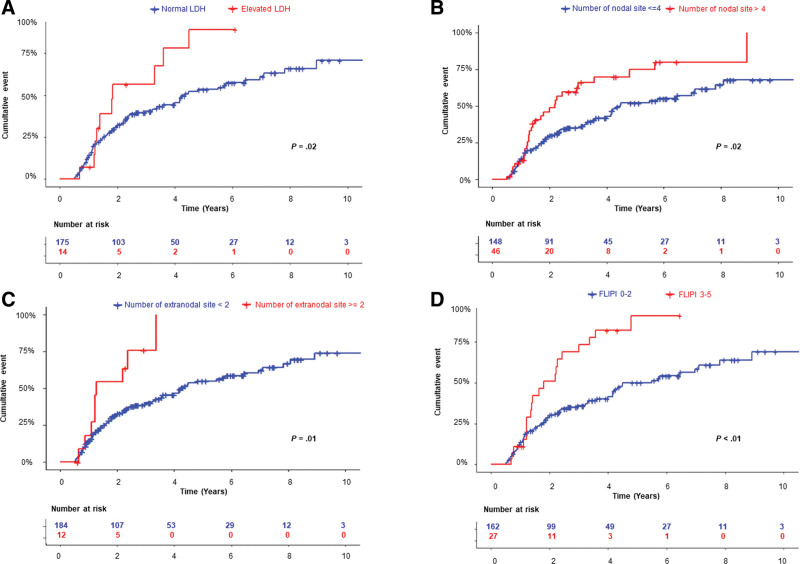
**Cumulative incidence of time to lymphoma treatment stratified by (A) LDH, (B) number of nodal groups, (C) number of extranodal involvement, and (D) FLIPI score.** FLIPI = Follicular Lymphoma International Prognostic Index; LDH = lactate dehydrogenase.

**Figure 3.**
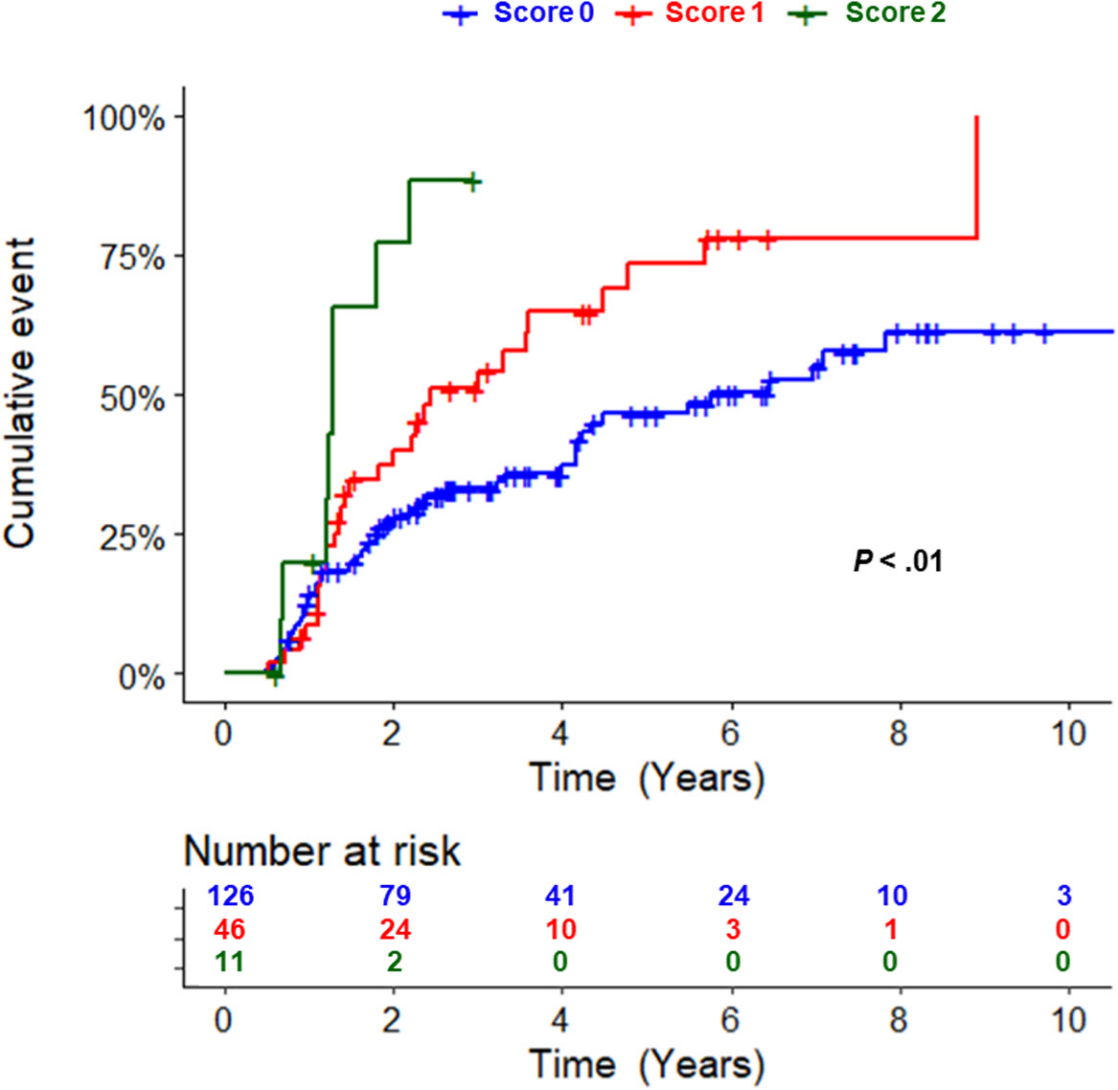
**Cumulative incidence of time to lymphoma treatment among FL patients based on LDH, number of nodal areas and number of extranodal involvement as risk factors.** One point was given to each variable. FL = follicular lymphoma; LDH = lactate dehydrogenase.

In multivariate analysis, independent factors of shorter TLT were elevated LDH (*P* = 0.02; HR = 2.2; 95% CI, 1.1-4.3), involvement of more than 4 nodal areas (*P* = 0.02; HR = 1.7; 95% CI, 1.1-2.8), and more than 1 extranodal site (*P* = 0.01; HR = 2.7; 95% CI, 1.2-5.7) (Table 2). The 2-year TLT rate was 57% (95% CI, 17.0%-77.2%) for patients with elevated LDH and 31% (95% CI, 23.8%-37.9%) for those with normal LDH (Figure 2A). The 2-year TLT rate was 46% (95% CI, 28.3%-59.5%) for patients with more than 4 nodal areas involved and 29% (95% CI, 21.6%-36.6%) for those with 4 or less (Figure 2B). The 2-year TLT rate was 54% (95% CI, 13.2%-76.2%) for patients with more than 1 extranodal site and 31% (95% CI, 24.6%-38.5%) for those with one or no extranodal site (Figure 2C). These 3 variables were available in 183 patients. Based on comparable HRs, 1 point was given to each variable. The median TLT was 5.8 years (95% CI, 4.2-10.7 years) for patients with no risk factor, 2.4 years (95% CI, 1.5-4.5 years) for patients with 1 risk factor, and 1.3 years (95% CI, 0.7-2.2 years) for patients with 2 risk factors (*P* < 0.01) (Figure 3 and Table 3). No patient presented with the 3 risk factors simultaneously. PFS and OS after first-line therapy according to risk groups were not statistically different (*P* = 0.72 and *P* = 0.61, respectively), with the limitation of a low number of events, in particular in the high-risk group, and the relatively short median follow-up after treatment (Suppl. Figure S2).

**Table 3:** Median Time to Lymphoma Treatment and Relative Risk of Progression According to Risk Group

Risk Group	Score	No. of Patients (%)	Median Time to Lymphoma Treatment (y)	HR	95% CI
Low	0	126 (69)	5.8	1.0	NA
Intermediate	1	46 (25)	2.4	1.9	1.2-2.9
High	>1	11 (6)	1.3	4.4	2.0-9.4

CI = confidence interval; HR = hazard ratio; NA = not applicable.

Given that 4 of the 5 variables of the FLIPI presented a *P* value <0.10 by univariate analysis, we applied it to our cohort of low-burden FL patients. Because patients with low-risk and intermediate-risk FLIPI had similar risk of progression, they were combined. The 2-year TLT rate was 51% (95% CI, 25.7%-67.8%) for patients with high FLIPI score and 29% (95% CI, 21.5%-35.8%) for patients with low and intermediate FLIPI score (*P* < 0.001). The median TLT was 4.5 years (95% CI, 4.0-7.1 years) for low/intermediate FLIPI score and 1.8 years (95% CI, 1.2-3.0 years) for high FLIPI score (Figure 2D). We also analyzed the prognostic role of the FLIPI score combined with involvement of more than 1 extranodal site into a multivariate analysis: both high FLIPI score (*P* = 0.004; HR = 2.2; 95% CI, 1.3-3.7) and more than 1 extranodal involvement (*P* = 0.04; HR = 2.8; 95% CI, 1.1-4.5) were independent predictors of inferior TLT. PRIMA-PI and FLIPI2 could not be analyzed due to missing data.

### PET-CT parameters

Among the 113 patients staged with PET-CT, 75 baseline PET-CT were available for quantitative parameters analysis. Median baseline TMTV was 40 cm^3^ (interquartile range [IQR]: 6–102) and mean baseline TMTV was 91 cm^3^. Median Dmax was 46 cm (IQR: 5–64) and median SDmax was 0.25 m^−1^ (IQR: 0.03–0.34). Regarding Dmax and SDmax, areas under the ROC curves for TLT were 0.59 (*P* = 0.03) and 0.62 (*P* = 0.01), respectively. ROC optimal cut-off value for SDmax was 0.32 m^−1^ with a sensitivity and specificity of 33% and 83%, respectively, for TLT. About two-third of the patients (n = 49 patients, 65%) had a TMTV greater than 14 cm^3^ and about a third of the patients (n = 23 patients, 31%) had a SDmax greater than 0.32 m^−1^. A high TMTV and a high SDmax were associated with Ann Arbor stage III/IV (94% versus 23%, *P* < 0.001, 100% versus 56%, *P* = 0.001, respectively), involvement of more than 4 nodal areas (51% versus 4%, *P* < 0.001, 81% versus 15%, *P* < 0.001), involvement of more than one extranodal site (18% versus 0%, *P* = 0.02, 30% versus 4%, *P* = 0.003), and high FLIPI score (37% versus 0%, *P* < 0.001, 48% versus 14%, *P* < 0.001). TMTV ≥ 14 cm^3^ was also associated with BM involvement (73% versus 0%, *P* = 0.004) (Suppl. Tables S2 and S3).

**Figure 4.**
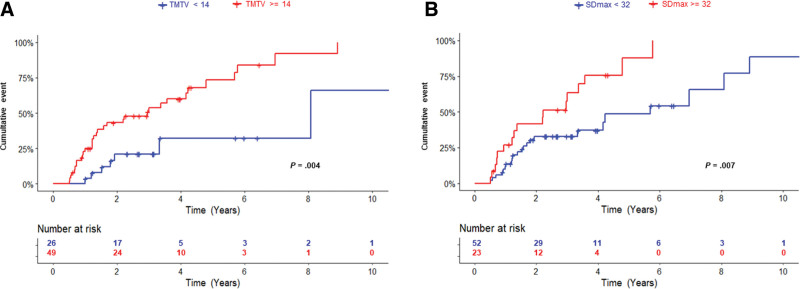
**Cumulative probability of time to lymphoma treatment among FL patients according to (A) TMTV and (B) SDmax.** FL = follicular lymphoma; SDmax = standardized Dmax; TMTV = total metabolic tumor volume.

In univariate analysis, a high TMTV (≥14 cm^3^) and a high SDmax (≥0.32 m^−1^) were significantly associated with a shorter TLT (*P* = 0.004; HR = 3.4; 95% CI, 1.5–7.6; *P* = 0.007; HR = 2.4; 95% CI, 1.3-4.7, respectively; Figure 4A and B).

In multivariate analysis, combining the 2 PET-CT parameters with the 3 risk factors previously identified (elevated LDH, >4 nodal areas, and >1 extranodal site), TMTV ≥14 cm^3^, elevated LDH, and more than one extranodal involvement were independent prognosticators for TLT (*P* = 0.02, *P* = 0.06, and *P* = 0.03, respectively) (Suppl. Table S4, model 1). In other models combining the 2 PET-CT parameters and one of the 3 “clinical” variables, TMTV remained independent predictor of shorter TLT whereas SDmax was not (Suppl. Table S4, models 2–4). In a model combining FLIPI and the 2 PET-CT parameters, only TMTV remained significant for TLT (Suppl. Table S4, model 5).

## DISCUSSION

The paradigm of deferring treatment is a widely used and safe strategy in indolent lymphomas and particularly in FL, applied for decades.^22,23^ Effectiveness of WW has been demonstrated in the chemotherapy era^8,9,24^ and then in the rituximab era.^7,15,16,25^ The use of single-agent rituximab in low-burden FL results in longer time to first chemotherapy but not in OS in selected and unselected populations of FL patients.^7,15,16,25^ As a result, a subset of FL patients, approximately 20%–40%, can be safely observed for a period of months to years (nearly 3 years) from initial diagnosis without impacting OS. Moreover, efficacy of first-line treatment does not seem to be affected by an initial WW phase, as well as the risk of HT. A recent study on 401 FL patients initially observed demonstrated that the likelihood of treatment initiation plateaued after 5 years of follow-up from diagnosis (12% for the next 5 years versus 43% at diagnosis).^26^ These data prompted us to search for potential risk factors of progression in FL initially observed to identify patients with high risk of progression in the first years.

In our report, the large majority of patients had favorable clinical and biological factors. Indeed, less than 10% of patients presented with altered ECOG PS, B symptoms, cytopenias, or elevated LDH. These findings are in line with previous reports and result in excellent OS.^15,16,26^ Identification of candidates for the WW approach is usually based on criteria reflecting high-burden tumor such as GELF criteria, with the exception of isolated LDH or ß2m. However, in routine practice, a significant proportion of FL patients managed by a WW strategy present with treatment initiation criteria as analyzed in the study by Khurana et al.^26^ The other important finding of this study was that presence of treatment initiation criteria at diagnosis (54% of patients) was not associated with increased therapy initiation rates, HT rates, or risk of lymphoma-related death.

In our study, the presence of one or more of the following parameters was associated with a higher risk of treatment initiation: elevated LDH, more than 4 nodal areas involved and more than 1 extranodal involvement. The involvement of more than 4 nodal groups and elevated LDH were already identified as risk factors of lymphoma treatment initiation/lymphoma-related death in 2 and 1 previous reports, respectively,^15,16^ and are part of the FLIPI score.^10^ Knowing that hemoglobin level <120 g/L and Ann Arbor stage III/IV were also associated with shorter TLT in univariate analyses, we explored the prognostic value of the FLIPI score. Patients with high FLIPI score (representing 14% of our cohort) had significant shorter time to progression compared with low/intermediate-risk FLIPI patients. In the study by Solal-Céligny et al,^15^ no correlation was found between FLIPI and FLIPI2 with TLT. A recent study has reported a dynamic use of the FLIPI score in initially observed FL patients, looking at changes in FLIPI score between diagnosis and during observation.^3^ FLIPI score increase was associated with inferior outcomes after first-line treatment but was not correlated with a shorter time to treatment initiation.

We were not able to analyze the prognostic value of FLIPI2 and PRIMA-PI due to missing data, mainly information on BM involvement. Indeed, only one-fifth of our cohort had BM examination at diagnosis. Compared with previous studies where BM biopsies were performed in 60%–95% cases,^3,26^ the lower rate in our report can be explained by the more recent inclusion period (2010–2020) with increasing use of PET-CT and reflects our center’s practices. The main drawback of BM biopsy is that it is an inadequate sampling of the entire BM. Staging by PET-CT has replaced the need for BM biopsy in diffuse large B-cell lymphoma (DLBCL) and Hodgkin lymphoma.^19,27^ Nakajima et al^28^ recently demonstrated that PET-CT improves the accuracy of staging FL compared with BM biopsy alone and above all has prognostic value on PFS and OS.

A new finding of our study was the association of more than 1 extranodal involvement with shorter time to progression in low-burden FL. This parameter has a known prognostic value in DLBCL, being one the variables of the international prognostic index, but is not part of prognostic scores used in FL. In the F2 study, extranodal involvement was present in 10% of patients managed by WW.^15^ Initial staging did not include PET-CT. In the present study, PET-CT enabled more accurate detection of extranodal involvement with 34% of patients (38/113) staged with PET-CT ± BM biopsy presenting with at least 1 extranodal involvement versus 9% of patients (8/88) staged without PET-CT. However, this variable retained its prognostic value to predict TLT when analyzing the population staged with PET-CT and the whole cohort. Using the 3 variables with statistical significance in multivariate analyses (elevated LDH, >4 nodal areas, and >1 extranodal involvement), we were able to identify patients who required treatment initiation within a median time of over 1 or 2 years for patients with 2 and 1 variables, respectively. Patients with 1 risk factor represent 25% of the cohort and those with 2 risk factors 6%. On the contrary, patients with no risk factors had a median time to progression of nearly 6 years. We were not able to identify PFS or OS differences after first-line treatment according to risk groups but our study was not powered to answer this question. Whether these variables could be used as criteria to initiate treatment remains to be studied with a much longer follow-up.

Given that these variables are clearly surrogates of tumor burden and lesion dissemination, we then analyzed some PET-CT parameters, that is, TMTV and the largest distance between 2 lesions (Dmax).^29^ The prognostic value of TMTV on baseline PET-CT has been largely described in various subtypes of lymphoma.^29–31^ Its value has been demonstrated in FL patients with high-tumor burden^13^ but very few data exist on low-burden FL patients managed by WW. SDmax is a more recent PET-CT parameter analyzed and its prognostic impact has been demonstrated in DLBCL^29^ and Hodgkin lymphoma.^32^ Interestingly, the optimal cut-off value for SDmax in our cohort (0.32 m^−1^) was the same as that found by Cottereau et al.^29^ Two recent studies have evaluated the role of quantitative PET-CT metrics in low-burden FL managed by WW.^21,33^ A study of 38 patients with FL initially observed found that SUVmax and total lesion glycolysis could predict shorter TLT,^33^ but not TMTV. Leccisotti et al^21^ retrospectively analyzed 54 FL patients managed by WW and staged with PET-CT. TMTV ≥14 cm^3^ was independently associated with shorter TLT. In our study, we confirmed the prognostic value of this threshold for TMTV. Moreover, TMTV ≥14 cm^3^ retained its independent prognostic value on TLT in several multivariate models combining quantitative PET-CT parameters and clinical variables or score. Further analyses on larger cohort of FL patients with baseline PET-CT will be needed to validate this finding.

Our study has some limitations. The identification of FL patients in this study was based on local databases and pathology lists, with potential selection biases. The retrospective nature of the study implies a lack of uniformity regarding follow-up visit and monitoring scans frequencies. Heterogeneity of staging can be underlined but is reflective of real-world practice. The relatively short follow-up of our cohort compared with other studies dealing with low-burden FL^3,25^ is not an obstacle considering the primary objective of our study to identify risk factors of early progression. On the contrary, it allowed us to analyze more recent patients managed with current treatment recommendations when progression occurs and a higher proportion of patients initially staged with PET-CT.

In conclusion, our work identifies elevated LDH, more than 4 nodal areas and more than 1 extranodal involvement as markers of shorter TLT in low-burden FL patients initially observed. Patients presenting with one or more of these parameters may require closer follow-up. Quantitative PET-CT metrics reflecting tumor dissemination and burden, particularly TMTV, seem promising in this subset of FL patients at the beginning of natural history. Future studies with more FL patients staged with PET-CT could help to better understand the prognostic impact of these parameters and the heterogeneity of outcomes observed in this population.

## AUTHOR CONTRIBUTIONS

CR, AD, and ED designed the study and wrote the manuscript; CR, LK, AD, and ED analyzed the data; all authors contributed to the interpretation of study data, critically reviewed the manuscript and approved the final version of the manuscript.

## DISCLOSURES

The authors declare no conflicts of interest.

## SOURCES OF FUNDING

The authors declare no sources of funding.

## Supplementary Material
