# Supplementary material for: Risk Factors of Progression in Low-tumor Burden Follicular Lymphoma Initially Managed by Watch and Wait in the Era of PET and Rituximab
Source: Hemasphere. 2023 Apr 26;7(5):e861. doi: 10.1097/HS9.0000000000000861 (PMC10146112; doi:10.1097/HS9.0000000000000861)
Supplement: Supplementary file 1 [file hs9-7-e861-s001.docx]

**Supplementary Appendix**

Supplement to: Rodier C, Kanagaratnam L, Morland D et al. Risk factors of progression in low-tumor burden follicular lymphoma initially managed by watch and wait in the era of PET and rituximab.

**Table of Contents**

Supplementary Table 1………………………………………………………………………….. 2

Supplementary Figure 1………………………………………………………………………..... 3

Supplementary Figure 2 ……….………………………………………………………………….. 4

Supplementary Table 2 ………………………………………………………………………….. 5

Supplementary Table 3 ………………………………………………………………………….. 6

Supplementary Table 4 ………………………………………………………………………….. 7

**Supplemental Table 1**. Type of extranodal involvement.

| Extranodal site | Total (n=201) | PET-CT at diagnosis  (n= 113) | No PET-CT at diagnosis  (n=88) |
| --- | --- | --- | --- |
| ≥ 1 extranodal site | 46 (23%) | 38 (34%) | 8 (9%) |
| Bone | 15 (7%) | 14 (12%) | 1 (1%) |
| Bone marrow | 14 (7%) | 11 (10%) | 3 (3%) |
| Digestive tract  Gastroduodenal  Small intestine  Colon  Rectum | 8 (4%)  3  1  2  2 | 7 (6%)  2  1  2  2 | 1 (1%)  1 |
| Leukemic phase | 7 (3%) | 5 (4%) | 2 (2%) |
| Spleen | 4 (2%) | 4 (4%) |  |
| Skin | 4 (2%) | 4 (4%) |  |
| Tonsil | 3 (1%) | 3 (3%) |  |
| Parotid | 2 (1%) | 2 (2%) |  |
| Lung | 2 (1%) | 2 (2%) |  |
| Pleural | 2 (1%) | 2 (2%) |  |
| Kidney | 2 (1%) | 2 (2%) |  |
| Pancreas | 1 (0.5%) | 1 (1%) |  |
| Breast | 1 (0.5%) | 1 (1%) |  |
| Adrenal | 1 (0.5%) | 1 (1%) |  |
| Conjunctiva | 1 (0.5%) | 1 (1%) |  |

**Supplemental Figure 1.** Overall survival (OS).

**
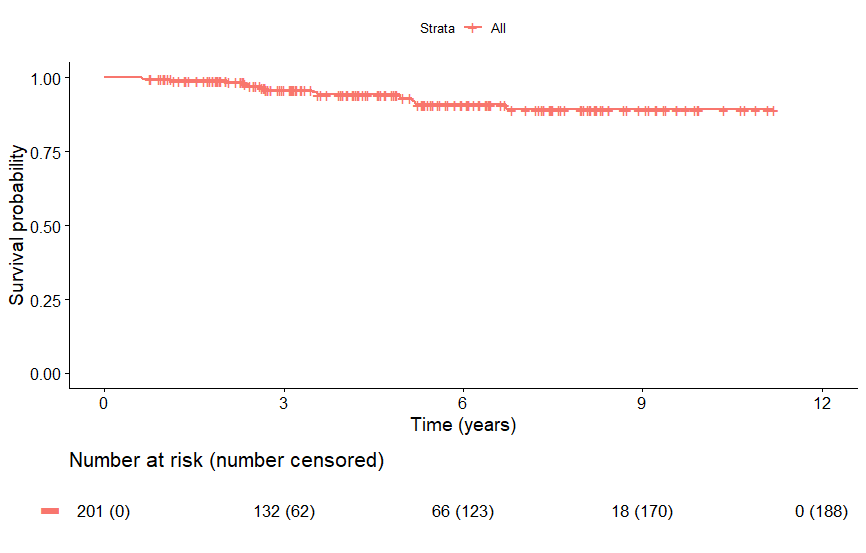
**

**Supplemental Figure 2.** (A) Progression-free survival after first-line treatment according to risk groups, (B) overall survival after treatment according to risk groups.


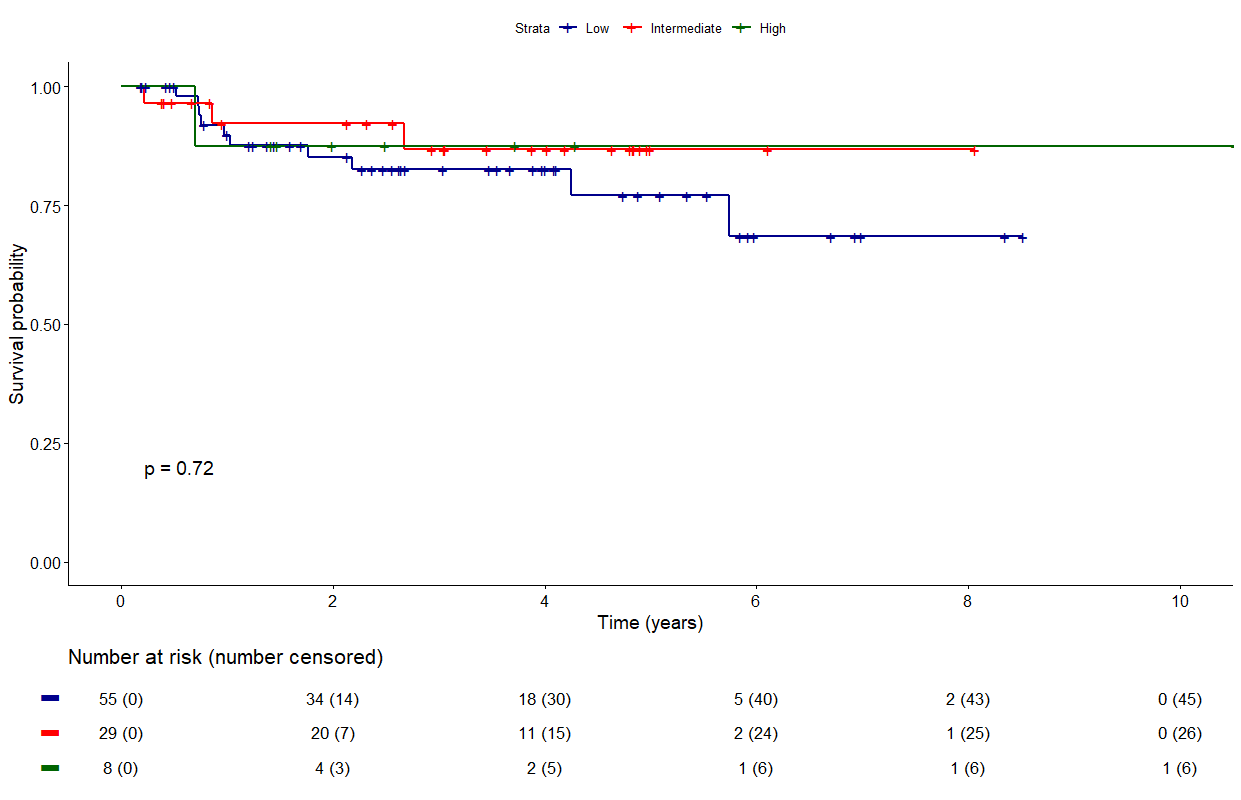
(A)


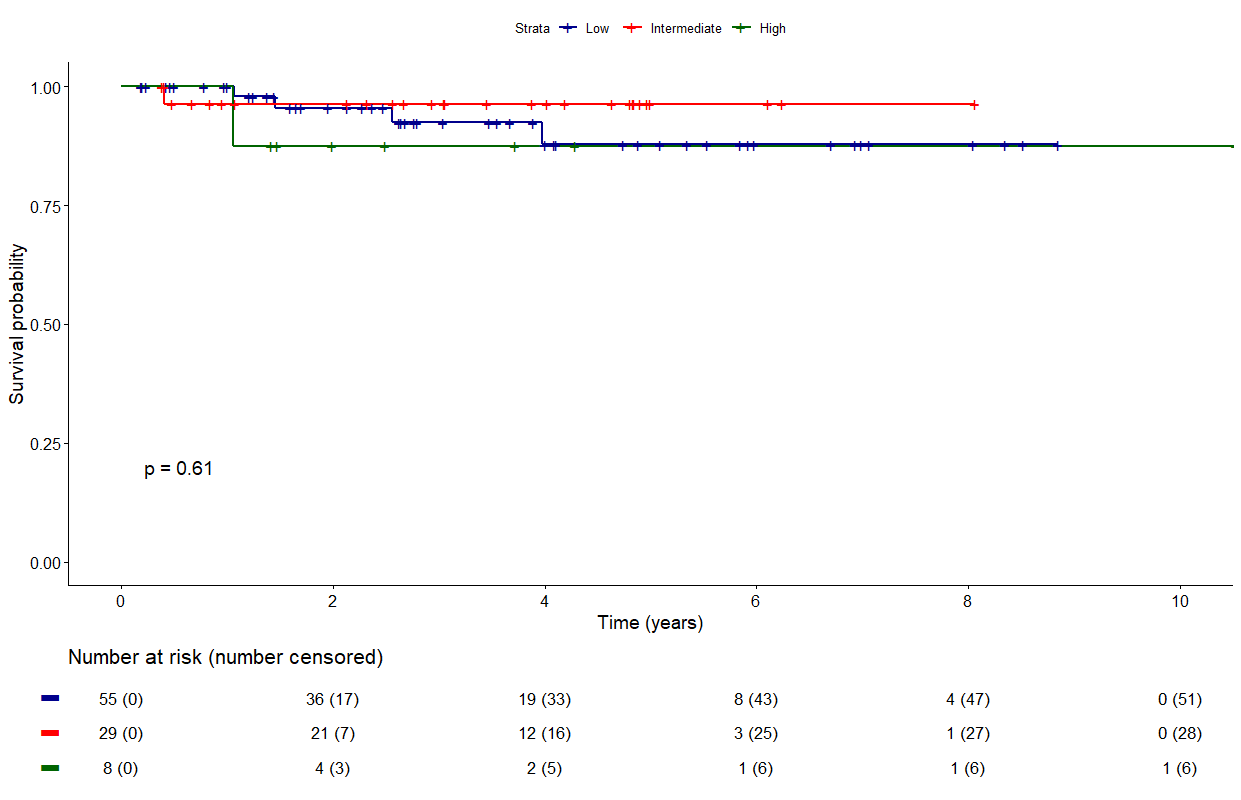
(B)

**Supplemental Table 2**. Patients’ characteristics according to TMTV.

| Characteristic | TMTV ≥ 14 cm^3^  (n = 49) | TMTV < 14 cm^3^  (n = 26) | *P*-value |
| --- | --- | --- | --- |
| Age > 60 years | 34 (69%) | 17 (65%) | 0.72 |
| ECOG performance status > 1 | 7 (14%) | 5 (21%) | 0.51 |
| B symptoms | 2 (4%) | 0 (0%) | 1 |
| Lymphopenia | 15 (31%) | 3 (12%) | 0.07 |
| Circulating lymphoma cells | 5 (11%) | 0 (0%) | 0.16 |
| Hemoglobin < 120 g/L | 0 (0%) | 0 (0%) | NA |
| Platelets < 150 10^9^/L | 4 (8%) | 1 (4%) | 0.66 |
| Elevated LDH | 3 (6%) | 2 (8%) | 1 |
| Elevated ß2m | 7 (15%) | 1 (5%) | 0.42 |
| Albumin < 3.5 g/dl | 1 (3%) | 0 (0%) | 1 |
| Number of nodal groups > 4 | 24 (51%) | 1 (4%) | < 0.0001 |
| Extranodal involvement ≥ 2 | 9 (18%) | 0 (0%) | 0.02 |
| Bone marrow involvement | 8 (73%) | 0 (0%) | 0.004 |
| Ann Arbor stage III/IV | 46 (94%) | 6 (23%) | < 0.0001 |
| FLIPI high-risk | 18 (37%) | 0 (0%) | < 0.0001 |

**Supplemental Table 3**. Patients’ characteristics according to SDmax.

| Characteristic | SDmax ≥ 0.32 m^-1^  (n = 23) | SDmax < 0.32 m^-1^  (n = 52) | *P*-value |
| --- | --- | --- | --- |
| Age > 60 years | 15 (65%) | 36 (69%) | 0.73 |
| ECOG performance status > 1 | 3 (13%) | 9 (18%) | 0.74 |
| B symptoms | 1 (4%) | 1 (2%) | 0.53 |
| Lymphopenia | 8 (35%) | 10 (20%) | 0.17 |
| Circulating lymphoma cells | 3 (14%) | 2 (4%) | 0.32 |
| Hemoglobin < 120 g/L | 0 (0%) | 0 (0%) | NA |
| Platelets < 150 10^9^/L | 1 (4%) | 4 (8%) | 1 |
| Elevated LDH | 0 (0%) | 5 (10%) | 0.31 |
| Elevated ß2m | 4 (18%) | 4 (9%) | 0.42 |
| Albumin < 3.5 g/dl | 0 (0%) | 1 (3%) | 1 |
| Number of nodal groups > 4 | 17 (81%) | 8 (15%) | < 0.0001 |
| Extranodal involvement ≥ 2 | 7 (30%) | 2 (4%) | 0.003 |
| Bone marrow involvement | 4 (57%) | 4 (36%) | 0.63 |
| Ann Arbor stage III/IV | 23 (100%) | 29 (56%) | 0.0001 |
| FLIPI high-risk | 11 (48%) | 7 (14%) | 0.0005 |

**Supplemental Table 4**. Multivariate analyses for time to lymphoma treatment (TLT) in 75 patients with baseline PET-CT available.

| Parameter | Multivariate Cox proportional hazard | |
| --- | --- | --- |
|  | **HR (95% CI)** | ***P*-value** |
| *Model 1* |  |  |
| TMTV ≥ 14 cm^3^  SDmax ≥ 0.32 m^-1^  Elevated LDH  Extranodal involvement ≥ 2  Number of nodal groups > 4 | 3.4 (1.2-9.6)  1.7 (0.7-4)  5.2 (1.6-16.8)  3.4 (1.1-10.1)  0.6 (0.3-1.5) | **0.02**  0.24  **0.006**  **0.03**  0.26 |
| *Model 2* |  |  |
| TMTV ≥ 14 cm^3^  SDmax ≥ 0.32 m^-1^  Elevated LDH | 2.6 (1.0-6.7)  1.8 (0.9-3.9)  4.8 (1.5-15.0) | **0.04**  0.11  0.007 |
| *Model 3* |  |  |
| TMTV ≥ 14 cm^3^  SDmax ≥ 0.32 m^-1^  Extranodal involvement ≥ 2 | 3.0 (1.2-7.7)  1.4 (0.6-2.9)  2.0 (0.8-5.0) | **0.02**  0.41  0.15 |
| *Model 4* |  |  |
| TMTV ≥ 14 cm^3^  SDmax ≥ 0.32 m^-1^  Number of nodal groups > 4 | 3.2 (1.3-8.1)  1.8 (0.8-3.9)  0.7 (0.3-1.5) | **0.01**  0.15  0.31 |
| *Model 5* |  |  |
| TMTV ≥ 14 cm^3^  SDmax ≥ 0.32 m^-1^  FLIPI high-risk | 2.8 (1.1-7.3)  1.5 (0.7-3.3)  1.1 (0.5-2.2) | **0.04**  0.25  0.95 |
